# Designing a primary care pharmacist-led review for people treated with opioids for persistent pain: a multi-method qualitative study

**DOI:** 10.3399/BJGPO.2023.0221

**Published:** 2024-08-07

**Authors:** Charlotte Woodcock, Nicola Cornwall, Lisa Dikomitis, Sarah A Harrisson, Simon White, Toby Helliwell, Roger Knaggs, Eleanor Hodgson, Tamar Pincus, Miriam Santer, Christian D Mallen, Julie Ashworth, Clare Jinks

**Affiliations:** 1 Centre for Musculoskeletal Health Research, School of Medicine, Keele University, Keele, UK; 2 Centre for Health Services Studies and Kent and Medway Medical School, University of Kent, Canterbury, UK; 3 Midlands Partnership University NHS Foundation Trust, Haywood Hospital, High Lane, Burslem, Stoke on Trent, UK; 4 School of Pharmacy and Bioengineering, Keele University, Keele, United Kingdom; 5 Division of Pharmacy Practice and Policy, School of Pharmacy, University of Nottingham, Nottingham, UK; 6 Primary Integrated Community Services Ltd, Nottingham, UK; 7 Leek Health Centre, Fountain Street, Leek, UK; 8 Department of Psychology, University of Southampton, Southampton, UK; 9 Primary Care Research Centre, University of Southampton, Southampton, UK

**Keywords:** pharmacists, analgesics, opioid, chronic pain

## Abstract

**Background:**

Opioids are frequently prescribed for persistent non-cancer pain despite limited evidence of long-term effectiveness and risk of harm. Evidence-based interventions to address inappropriate opioid prescribing are lacking.

**Aim:**

To explore perspectives of people living with persistent pain to understand barriers and facilitators in reducing opioids in the context of a pharmacist-led primary care review, and identify review components and features for optimal delivery.

**Design & setting:**

A multi-method qualitative study undertaken in the primary care setting in the UK.

**Method:**

Adults with experience of persistent pain and taking opioids participated in semi-structured interviews (*n* = 15, 73% female) and an online discussion forum (*n* = 31). The Theoretical Domains Framework (TDF) provided a framework for data collection and thematic analysis, involving deductive analysis to TDF domains, inductive analysis within domains to generate sub-themes, and sub-theme comparison to form across-domain overarching themes. The behaviour change technique taxonomy (v1) and motivational behaviour change technique classification system were used to systematically map themes to behaviour change techniques to identify potential review components and delivery features.

**Results:**

Thirty-two facilitator and barrier sub-themes for patients reducing opioids were identified across 13 TDF domains. These combined into the following six overarching themes: learning to live with pain; opioid reduction expectations; assuming a medical model; pharmacist-delivered reviews; pharmacist–patient relationship; and patient engagement. Sub-themes mapped to 21 unique behaviour change techniques, yielding 17 components and five delivery features for the proposed PROMPPT review.

**Conclusion:**

This study generated theoretically informed evidence for design of a practice pharmacist-led PROMPPT review. Future research will test the feasibility and acceptability of the PROMPPT review and pharmacist training.

## How this fits in

There is a need to develop evidence-based primary care interventions to address overprescribing of opioids for persistent non-cancer pain. Best practice guidance recommends the regular review of patients prescribed long-term opioids for persistent non-cancer pain, and advises gradual reduction of opioids if treatment goals are not met. This study identified facilitators of and barriers to patients reducing opioids in the context of a pharmacist-led review in primary care. The findings were mapped to behaviour change techniques to inform the design of a practice pharmacist-led review for patients prescribed opioids for persistent pain (PROMPPT review; Proactive clinical Review of patients taking Opioid Medicines long-term for persistent Pain led by clinical Pharmacists in primary care Teams) for testing in a feasibility study, ahead of a full-scale randomised controlled trial.

## Introduction

Persistent pain, or pain lasting ≥3 months and not caused by cancer, affects around 43% of UK adults, with 10%–14% reporting disabling pain that is moderately-to-severely limiting.^1^ Opioid prescribing for persistent pain has increased markedly during the past 20 years,^2,3^ despite a lack of evidence for long-term effectiveness and growing evidence of harms.^4,5^

Best practice guidance recommends regular review of patients prescribed long-term opioids for persistent pain, and gradual reduction of opioids if treatment goals are not met.^6,7^ Most opioid prescribing for persistent pain occurs in primary care and GPs report barriers to routinely reviewing patients, citing a lack of training, resources, and time.^8^ There has been a recent expansion in pharmacists working in GP practices in UK primary care. Practice pharmacists’ expertise in medicines optimisation should make them well-placed to review patients prescribed opioids for persistent pain.^9–12^

This study forms part of a larger research programme called PROMPPT (Proactive clinical Review of patients taking Opioid Medicines long-term for persistent Pain led by clinical Pharmacists in primary care Teams). The programme aims to develop a proactive primary care review for patients prescribed opioids for persistent pain (called ‘PROMPPT review’ herein) delivered by practice pharmacists (called ‘pharmacist’ herein).

Intervention development is a dynamic and iterative process based on evidence and understanding of the target behaviour of reducing opioids.^13–15^ Although previous research identifies potential patient barriers to reducing opioids (for example, benefits of opioids outweigh risks,^16^ fear of increased pain,^17^ lack of effectiveness of non-pharmacological options),^18^ there is limited evidence within the context of primary care. Using a person-based approach,^15^ this study aims to identify barriers and facilitators to people with persistent pain reducing opioids in the context of a pharmacist-led review in primary care (that is, PROMPPT review); to use this information to identify potential components for a PROMPPT review; and to determine key features for its optimal delivery.

## Method

### Design

A multi-method qualitative study comprising of interviews and an online discussion forum was conducted. Qualitative data collection and analysis was informed by the Theoretical Domains Framework (TDF).^19^ The TDF is used for developing theory-informed interventions and has 14 domains to identify facilitators and barriers of behaviour change.^20^ TDF domains are linked to behaviour change techniques (BCTs)^14,21^ and provide a systematic approach for identification of potential PROMPPT review components through mapping to BCT taxonomies.^14,22^

### Semi-structured interviews

From September 2019–October 2019, adults (aged >18 years) prescribed any opioid analgesic for ≥6 months for persistent pain were recruited from two GP practices in the West Midlands, UK. To gain wide-ranging perspectives, patients were purposively sampled according to sex and strength of opioid medicine (weak, intermediate, or strong) based on published categorisation for prescribed analgesics in primary care (Table 1).^23^

**Table 1.** Categorisation of patients by opioid strength based on a hierarchy of analgesic potency arising from a consensus study of UK GPs^23^

Weak	Intermediate	Strong
Co-codamol 8 mg/500 mg	Codeine 30 mg	Morphine
Co-codamol 15 mg/500 mg	Co-codamol 30 mg/500 mg	Oxycodone
Codeine 15 mg	Dihydrocodeine 30 mg	Fentanyl
Codeine 20 mg	Buprenorphine patch ≥15 mcg/hour	Tapentadol
Co-dydramol 10 mg/500 mg	Buprenorphine sublingual 400 mcg	Diamorphine
Co-dydramol 20 mg/500 mg	Tramadol >37.5 mg	Hydromorphone
Dihydrocodeine 20 mg	Pethidine	Dipipanone
Co-proxamol 32.5 mg/325 mg	Pentazocine	Dextromoramide
Tramadol 37.5 mg/500 mg	Meptazinol	
Buprenorphine patch 5 or 10 mcg/hour		
Buprenorphine sublingual 200 mcg		

Interview guides, informed by the TDF, were drafted with public contributors and aimed to explore experiences of persistent pain, pain management strategies (including opioids), and views on a proposed PROMPPT review (Supplementary Topic Guide S1).

Interviews were conducted by NC in-person or via telephone, according to participant preference, and digitally audiorecorded. Recruitment stopped when data saturation had been reached.^24^ Participants were aware they would be interviewed about their regular medicines and what is important to them to help design a pain medication review. Participants were offered a £10 voucher to thank them for their contribution to the study.

### Online discussion forum

From October 2019–December 2019, adults (aged >18 years) with experience of opioids for persistent pain were invited to register and contribute to a bespoke online discussion forum via posters (electronic and paper) displayed in GP practices, pain services, community pharmacies across the West and East Midlands and Wessex in the UK, as well as via online posts and paid advertisements using social media (Twitter [now called X] and Facebook). The online discussion forum was developed by the research team using Discourse,^25^ in conjunction with patient and public user testing.^26^

Ten topics for discussion were published on the forum over 11 weeks (Supplementary Topic Guide S2). The first six topics were generated by the research team, guided by TDF domains and input from public contributors. The four remaining topics drew on preliminary themes identified from interview data and stakeholder discussions with patients, pharmacists, general practice managers, GPs, practice nurses, physiotherapists, psychologists, and addiction specialists. Each topic opened with an audiovisual animation to introduce the main question for discussion, below which participants could post comments and questions, and react to other participants’ responses. There was also a ‘Community Hang Out’ page where participants could discuss additional topics. The discussion forum was moderated at regular intervals between 8am and 10pm, Monday to Sunday, to ensure ethical guidelines were upheld. Discussion threads were facilitated by CW, providing prompts and probes to explore participant posts in greater depth and invite other participants into the discussion. Facilitation was supported by regular meetings with LD and discussions with the wider research team.

Research team members collecting data were experienced post-doctoral qualitative researchers. None of the research team knew the participants before their involvement in the study.

### Data preparation and analysis

Interview recordings were transcribed verbatim, anonymised, and checked for accuracy. Discussion forum posts were anonymised, and forum user IDs replaced with de-identifying codes.

A three-phase analysis process examined the data for facilitators and barriers to reducing opioids and valued intervention delivery features for a PROMPPT review. First, deductive analysis of the data was conducted where text segments were coded and indexed to relevant domains of the TDF framework. Researchers with expertise in applied health research (CJ), psychology (NC, CW), pharmacology (SW), and general practice (TH) independently completed this deductive process for at least one of three transcripts following initial stages of framework analysis^27^ of familiarisation (that is, reading and re-rereading of transcripts), coding (that is, identifying segments of text relevant to the research question), and indexing segments of text to TDF domains (that is, organising codes to relevant domains). Meetings were held to discuss analytical decisions with additional viewpoints from two clinical academics specialising in pain management (JA, SH) to ensure no one disciplinary perspective dominated.^28^ Following discussions, a refined framework^20^ was used by three researchers (NC, EH, CW) to deductively index remaining data with regular meetings to ensure a robust approach. NVivo software (version 12) was used to aid data management. Second, data segments indexed to each TDF domain were inductively analysed to generate domain-specific sub-themes. Third, sub-themes were compared and related sub-themes brought together to form overarching themes.^28,29^ These inductive analytical phases were carried out by CW with regular critical discussion with CJ and presented to the wider research team.

### Theory-based mapping to behaviour change techniques

Facilitator and barrier sub-themes were used to identify BCTs for the PROMPPT review. This process drew on the taxonomy of behaviour change techniques (BCTTv1)^22^ and the classification system for motivational behaviour change techniques (MBCTs).^30^ BCTTv1 links to TDF domains via expert consensus^21^ and provides a common terminology for identifying an intervention’s ‘active ingredients’ for change. MBCTs are underpinned by self-determination theory^31^ that states intrinsic motivation to engage with an intervention depends on perceived fulfilment of three universal basic psychological needs of autonomy (for example, decision to reduce opioids is self-endorsed), competence (for example, feel in control and confident in making an opioid reduction), and relatedness (for example, feel accepted, respected, and sense of connectedness with the pharmacist supporting an opioid reduction).^30^

### Patient and public involvement (PPI)

Members of Keele University School of Medicine’s PROMPPT Research User Group (RUG), with lived experience of persistent pain, contributed to the design of data collection methods. For interviews, PPI members identified topics to guide interview questions (for example, attitudes towards opioids, experiences of medication reviews). For the discussion forum, PPI members advised on participant recruitment and engagement strategies as well as design features of audiovisual animations. Members also tested the forum’s usability before data collection.^26^ The GRIPP2 short-form checklist was completed for reporting PPI.^32^

## Results

From 120 study invitations, 22 consent-to-contact forms were received requesting further study information. Seventeen reply forms agreed to arrange an interview, from which 15 interviews (73% with female participants) were conducted in-person or by telephone according to participant preference (mean length of 37 minutes) See Table 2 for demographics. Thirty-one participants posted a total of 160 comments to the online discussion forum. Comments ranged in length between 19 and 2143 words.

**Table 2.** Participant demographics of people living with persistent non-cancer pain (*n* = 15 interviews)

		**Opioid strength**			**Total**
**Sex**	**Age range (mean) years**	**Weak**	**Intermediate**	**Strong**	
Male	55–83 (68.75)	1	1	2	4
Female	54–87 (70.73)	2	4	5	11
All	54–87 (70.20)	3	5	7	15

Opioid strength based on published categorisation for prescribed analgesics in primary care.^23^

Six overarching themes, grouping 32 sub-themes across 13 TDF domains, were identified and describe the complex interaction of facilitators and barriers to reducing opioids in the context of a pharmacist-led review in primary care namely, learning to live with pain, opioid reduction expectations, assuming a medical model, pharmacist-delivered reviews, pharmacist–patient relationship, and patient engagement (see Table 3 and Supplementary Tables S3 and S4).

**Table 3.** TDF domains, facilitator and barrier sub-themes, and overarching themes for patients reducing opioids in the context of a PROMPPT review

TDF domain	Sub-theme	F^a^	B^b^	Overarching theme
Knowledge	Knowing about and managing pain	✓	✓	Learning to live with pain
Behavioural regulation	Self-regulating pain management	✓	✓	
Environmental context and resources	Accessible evidence-based resources	✓		
Social influences	Social support	✓	✓	
Social or professional role and identity	Changing identities	✓	✓	
Goals	Live better with pain	✓		
Knowledge	Knowing about reducing opioids	✓	✓	Opioid reduction expectations
Behavioural regulation	Monitoring for quick effectiveness of opioid reduction	✓	✓	
Beliefs about capabilities	Unable to cope with an opioid reduction		✓	
Beliefs about consequences	Consequences of reducing opioids	✓	✓	
Intentions	Intention to reduce	✓	✓	
Emotions	Anxious about reducing opioids		✓	
Reinforcement	Avoid withdrawal		✓	
	Reduce if potential benefits perceived	✓		
Social influences	Prescribed by healthcare professional		✓	Assuming a medical model
Reinforcement	Opioids are necessary		✓	
	Left on repeat prescription		✓	
Knowledge	Pharmacist knowing about and managing pain within primary care	✓		Pharmacist-delivered reviews
Skills	Patient-centred shared decision making	✓		
Social influences	Patient–clinician relationship	✓	✓	Pharmacist–patient relationship
	Supportive point of contact for pain management	✓		
Knowledge	Patient knowledge of PROMPPT review	✓		Patient engagement
Environmental context and resources	Accessibility of a PROMPPT review	✓	✓	
Beliefs about capabilities	Able to discuss experiences of pain, medicines, and management	✓		
Beliefs about consequences	Wide-ranging benefits	✓		
	PROMPPT review concerns		✓	
	Provide a pharmacological solution		✓	
Intentions	Intention to engage in a PROMPPT review	✓	✓	
Goals	Find a pharmacological solution		✓	
	Increase understanding of pain and medicines	✓		
Optimism	Optimistic a PROMPPT review will be helpful	✓		
	Uncertain of personal relevancy of a PROMPPT review		✓	

^a^F = facilitator. ^b^B = barrier.

### Learning to live with pain

Learning to live with pain reflects the (often long) journey many people have experienced in learning how to best manage, and live with, pain. Participants said their care involved multiple healthcare professionals (for example, GP, physiotherapist, pain consultant, or clinical psychologist) with varying degrees of satisfaction. Many spoke of exploring different pharmacological options, prescribed and non-prescribed, to find out what best suits them. For some, the strength of their opioids escalated over time, or modes of administration altered.

*'It has taken me all the years since my injury to find a pain routine that works for me. But it still involves tramadol. My dose has never increased, nor have I had to change painkillers, but I did have to switch to modified release to try and stop the peaks and troughs.'* (ODF [online discussion forum] 12)

Such comments suggest ‘pain routines’ develop over time and encompass constant monitoring and responding to fluctuating pain levels. Despite these routines, participants told us they ‘*don’t like*’ (I [interview] 01, 02, 04, 12, 15, 20, 22) or even ‘*hate*’ (ODF20, 31, 39) taking their medicines and some questioned their effectiveness. These negative perceptions of opioids were discussed in relation to experiencing adverse side effects (for example, constipation fatigue), learning about long-term risks (from healthcare providers, the news, or scientific articles), and not wanting to rely on medication. Despite these views, the belief opioids are a necessary part of pain management prevails:

*'I don’t want to have them. I’ve never been a person that wants to take pills … but I know I’ve got to. I’ve accepted that I have to.'* (I03)

In conjunction with opioids, many participants talked about trying non-pharmacological approaches for pain management including physical activity classes (for example, tai chi, yoga), self-directed activity (for example, walking), physiotherapy exercises, soothing strategies (for example, hot showers or hydrotherapy), and complementary therapies (for example, arnica or magnesium). Participants spoke about the value non-pharmacological strategies have in compensating for, or replacing the role of, medication as well as having additional psychological and social benefits.

*'… walking has been very important for both physical and mental health. Yoga is awesome. Ballet is great fun. And the social aspects are great as well.'* (ODF05)

Participants, whose journey involved stopping opioids, spoke of changes to their knowledge of pain, acceptance of its persistent nature, finding new (non-pharmacological) ways to manage pain, and understanding what this means for their sense of self. For example, one participant explained ‘*due to the nature of my health my outlook on the world is vastly different to the norm*’ (ODF24). Participants told us making changes to how they manage their pain was sometimes challenging but was made possible by drawing on multiple resources (for example, mobile apps, online information from credible sources, trusted healthcare professionals, or social support).

### Opioid reduction expectations

Participants’ expectations of reducing opioids seemed to vary. Some participants said their opioids helped manage their pain and questioned the reason for reducing. One participant said, ‘*don’t fix if it’s not wrong’* (I15). Some participants shared failed attempts to reduce opioids experiencing ‘*crisis in withdrawal*’ (ODF05) and voiced concerns that any reduction would lead to compromised functionality and deterioration of other health conditions.

*'... every time I leave it off I’m just in that much pain it isn’t worth it, it’s either have a life or not have a life.'* (I07)

In contrast, participants willing to reduce opioids anticipated potential benefits (for example, fewer adverse side effects). Nevertheless, these participants also expressed anxieties around the process. Some told us they had been taking opioids ‘*so long*’ (I04) reducing was an unknown and they feared not having anything else for their pain or suffering withdrawal. Participants expressed caution and told us if they perceived pain to worsen they would reinstate their opioids.

*'... if I reduced it and it wasn’t working, then you just start taking it again don’t you?'* (I22)

Some participants who had reduced opioids spoke about (sometimes surprising) positive outcomes (for example, less pain, improvements to quality of life).

*'I started reducing my morphine .... when I had dropped to 90 mg, I noticed I was in less pain ....! I continued .... maybe a bit quicker than I should have because I was excited.'* (ODF02)

These quotes highlight how participants might closely monitor how reducing opioids impacts pain and how this may affect engagement with a tapering process.

### Assuming a medical model

Some participants appeared to adopt a medical model for managing pain, whereby their focus was on seeking pain relief, primarily through prescribed medication. Several participants told us opioids were necessary as they had been recommended by healthcare providers, provided some pain relief, and there seemed to be no alternative. One participant said they were ‘*stuck*’ (I19) with opioids, and others said they had ‘*no choice*’ (I02, 22) but to continue them.

Interactions with healthcare professionals also seemed to reinforce this pharmacological model as one patient recounted being told they would ‘*always have to rely on drugs*’ (I02). Where medicines were left on repeat prescription this was viewed by some as a sign to continue their use.

*'.... at the moment the hip pain has gone but I’m still on a repeat prescription for this co-codamol so I take it.'* (I09)

In contrast, participants who adopted a more holistic view of pain management viewed opioids on repeat prescriptions as a consequence of inactivity by the medical profession. One participant told us ‘*you’re just left*’ (I11) and another expressed that ‘*chronic pain patients are left to linger and slowly deteriorate by the medical system*’ (ODF05).

### Pharmacist-delivered reviews

Participants told us that pharmacists delivering PROMPPT reviews needed up-to-date knowledge about persistent pain, the physical and psychological impact of pain, and its appropriate management. Participants recognised pharmacists’ expertise in medicines but felt knowledge around non-pharmacological interventions, support services, and resources was also key.

*'Up to date and sustained development of their knowledge of pain management and routes they can use to resources that support patients.'* (ODF06)

Drawing on previous experiences, participants offered examples of what they would find off-putting or prefer not to happen in a PROMPPT review; for example, when processes felt externally imposed, patients felt like a nuisance, with no opportunity to explain what living with pain is like for them. Instead, participants expressed a preference for a person-centred collaborative approach where pharmacists are ‘*prepared to listen*’ (I04), ‘*use the information they’re getting from* [patients]’ (I15), and come to ‘*an agreed outcome or goal*’ (ODF37).

### Pharmacist–patient relationship

Participants highlighted the importance of the pharmacist–patient relationship. Previous negative interactions with healthcare professionals left participants feeling misunderstood, disbelieved, and stigmatised with one participant saying their ‘*confidence and trust in medics has been destroyed*’ (ODF05). Instead, participants wanted to ‘*build up a rapport*’ (I14) with healthcare professionals based on trust, empathy, and compassion, but recognised that developing rapport can take time and depends on continuity of care. Other reported facilitators of forming good patient–pharmacist relationships included pharmacists having more time than GPs, being recommended by trusted individuals (for example, GP, friends, or family) and patients informed about pharmacists’ expertise and qualifications.

### Patient engagement

Participants told us about facilitators for engaging in a PROMPPT review and include knowing the purpose of the review, having confidence to discuss experiences of pain, and holding positive outcome expectations (for example, an opportunity to discuss and alleviate any concerns about their medicines). Several participants expressed optimism that a review would be helpful, provide an opportunity to discuss their pain, learn more about their condition and medication, and lead to improvements in pain management, pain relief, psychological wellbeing, and quality of life.

*'I think it would achieve peace of mind … and emotionally I think it would be good … to be able to get it off your chest and talk to somebody who knows and who understands.'* (I13)

Some participants felt patients may not engage with the review if they believed it was a money-saving exercise, or in knowing alternative medications do not exist might consider the review as having little to offer.

'*We know GPs meet to discuss patients on pain medication, as I was warned by one in my practice that the head GP …* [they were] *bringing me up as an example of who costs too much.'* (ODF57)

Participants also spoke about the importance of making the PROMPPT review accessible and fit-for-purpose. Some participants could not always get to their general practice owing to relying on others for travel or because pain made travelling difficult. They felt flexible delivery of PROMPPT reviews (for example, in-person or remote) was desirable. Participants highlighted difficulties getting appointments and lack of time in appointments as other potential barriers to address.

### PROMPPT review components and delivery features

Drawing on the TDF domains and sub-themes within each overarching theme, we identified 21 behaviour change techniques (10 BCTs and 11 MBCTs), guided by expert consensus where available,^21^ to address barriers and facilitators for reducing opioids, and optimise delivery of the proposed PROMPPT review (Supplementary Figures S1 and S2).^14,22,30^ All TDF domains were included in this process except Social or professional role or identity*,* for which experts could not reliably allocate BCTs during a consensus-rating exercise meaning no BCTs were recommended for supporting change in this domain.^21^ Translation of BCTs and MBCTs into 17 proposed PROMPPT review components and five delivery features was discussed with the research team.

## Discussion

### Summary

This study provides theoretically grounded qualitative evidence informing the development of a pharmacist-led review within primary care (PROMPPT review), to support opioid tapering, where appropriate, for patients with persistent pain. Six overarching themes, representing key considerations for developing the PROMPPT review, were generated namely the following: learning to live with pain; opioid reduction expectations; assuming a medical model; pharmacist-delivered reviews; pharmacist–patient relationship; and patient engagement. From these findings, we used established behaviour change technique taxonomies (BCTTv1^22^ and MBCT classification system)^30^ to identify potential PROMPPT review components and delivery features.

### Strengths and limitations

A key strength of this study is its robust systematic approach in using an established theoretical framework, by a multidisciplinary research team, to understand the views of people living with persistent pain of a new review in the context of primary care. This rigorous process is important to ensure comprehensive consideration is given to the attitudes, beliefs, and needs of those who an intervention is intended for, in order to identify intervention components and delivery features that seem most acceptable and feasible.^15,33^ This approach provides a framework for guiding the analysis of future evaluations and implementation of the PROMPPT review using identified facilitators and barriers within TDF domains across overarching themes.

Another main strength of this study was the multi-method approach that provided people living with persistent pain different options for participation. The inclusion of a bespoke online discussion forum provided an alternative, innovative method of data collection,^26^ allowing participants to participate at a time and place most comfortable for them.^34^ Flexibility of participation is particularly important for those with chronic conditions, where unpredictable symptoms can be a barrier to participating in research.^35^ Another benefit of the discussion forum was in reaching people who had successfully stopped taking opioids. Including these voices is often more difficult than those currently seeking treatment and identifiable through medical records^36^ yet they provide important insights into potential facilitators for reducing opioids.

A further strength of the study was the extensive role of PPI in the development and design of the online discussion forum.^26^ PPI user testing suggested the platform was accessible, easy to navigate and use. In future, it may be beneficial to also involve PPI during the process of data collection and contribute to facilitation strategies of participant online discussions as well as analysis.

One limitation of the study is a lack of consideration of how patients’ experiences in any specialist services they access for persistent pain may impact their perception of the PROMPPT review. Another weakness of this study is the limited information collected about participant characteristics. For the interviews only sex, age, and opioid strength were collected. We decided not to systematically collect demographic information of online discussion forum participants to promote anonymity; an important factor for feeling empowered online, reducing feelings of vulnerability, and facilitating opening up and posting of comments.^37^ Although we documented participants’ sex when this was volunteered in forum posts, limited demographic information means that conclusions cannot be made about the diversity of perspectives and the extent to which voices from seldom heard or underserved communities were included. It was hoped the discussion forum would overcome barriers (for example, minimise researcher–participant power in-balance);^38^ however, the extent to which this was achieved cannot be assessed.

### Comparison with existing literature

Previous research has explored patient facilitators and barriers to opioid tapering. For example, qualitative research and syntheses have reported that patients believe there is no alternative to opioids,^29^ take opioids reluctantly,^39^ and view them as both a salvation and a curse.^40^ Our study echoes these findings and suggests people perceive opioids as a necessary part of established pain routines and, for some, as an enabler for living better with pain. This study considers such barriers within and across broader overarching themes that summarise multiple relating domains of influence such as patient beliefs, availability of resources, and social factors. For example, the overarching theme of learning to live with pain encapsulates personal journeys of finding acceptable ways to live with pain and establish pain management routines, which often include opioids. The involvement of opioids in these routines is strengthened when patients assume a medical model for pain management and hold negative opioid reduction expectations. These learning journeys and associated beliefs are reminiscent of ‘pain stories’. Previous research indicates the importance of respecting and validating patient pain stories, connected beliefs and associated emotions, when a potential change to pain management is to be broached.^41^

Previous research underlines the importance of the patient–clinician relationship for discussions around persistent pain and reducing opioids as there is potential for disagreements.^42^ Our study identified the pharmacist–patient relationship as a facilitator of meaningful discussions around pain management, particularly when pharmacists are skilled in active listening, expressing empathy and compassion. Although some of these behaviours overlap with principles of shared decision making, Matthias and Henry argue that shared decision making can be delivered with a narrow focus (for example, discussing pros and cons, risks and benefits of opioids) and does not always emphasise an environment of care, concern, and mutual trust.^43^ Many participants in our study did not know their practice pharmacist. This may present a challenge for pharmacist-delivered reviews and it is likely the development of a therapeutic pharmacist–patient relationship needs to be supported to promote patient engagement.

### Implications for research and practice

This study provides a theoretical and systematic person-based approach to identifying potential components and delivery features for a pharmacist-led PROMPPT review using evidence about facilitators of and barriers to patients reducing opioids. Since this work was completed, NHS England has published medicines optimisation guidelines for dependence-forming medicines in the form of a framework for action.^44^ Structured medication reviews (SMRs) are a key part of this framework and practice pharmacists are likely to lead SMRs. Proposed components and delivery features for the PROMPPT review are consistent with these recommendations. For example, the proposed delivery feature 2 ‘*pharmacist adopts a person-centred approach using shared decision-making skills*’ (Supplementary Figure S2) reflects action 1 of the framework: Personalised care and shared decision making.

The proposed components and delivery features for a PROMPPT review were taken forward for co-designing an intervention with key stakeholders taking into account the context of primary care and findings from our other intervention development work about potential acceptability of PROMPPT.^33^ Findings from this study also highlight potential training needs for practice pharmacists and informed guiding principles for the PROMPPT review. Future research will consider how pharmacists deliver the PROMPPT review to support patient engagement, confidence, and motivation to make a change; test the feasibility and acceptability of delivering the PROMPPT review in practice; and evaluate its clinical and cost-effectiveness in a cluster randomised controlled trial.

## Data

The dataset relied on in this article is available from the corresponding author on reasonable request and where participant consent allows.
